# Plasmatic Concentrations of ADMA and Homocystein in Llama (*Lama glama*) and Regulation of Arginase Type II: An Animal Resistent to the Development of Pulmonary Hypertension Induced by Hypoxia

**DOI:** 10.3389/fphys.2018.00606

**Published:** 2018-05-29

**Authors:** Vasthi López, Fernando A. Moraga, Anibal J. Llanos, German Ebensperger, María I. Taborda, Elena Uribe

**Affiliations:** ^1^Laboratorio de Metabolismo de Aminoácidos e Hipoxia, Departamento de Ciencias Biomédicas, Universidad Católica del Norte, Coquimbo, Chile; ^2^Laboratorio de Fisiología, Hipoxia y Función Vascular, Departamento de Ciencias Biomedicas, Facultad de Medicina, Universidad Católica del Norte, Coquimbo, Chile; ^3^Laboratorio de Fisiología y Fisiopatología del Desarrollo, Departamento de Ciencias Biomédicas, Universidad de Chile, Santiago, Chile; ^4^Laboratorio de Enzimología, Departamento de Bioquímica y Biología Molecular, Universidad of Concepción, Concepción, Chile

**Keywords:** llama, ADMA, pulmonary hypertension, homocysteine, arginase

## Abstract

There are animal species that have adapted to life at high altitude and hypobaric hypoxia conditions in the Andean highlands. One such species is the llama (*Lama glama*), which seem to have developed efficient protective mechanisms to avoid maladaptation resulting from chronic hypoxia, such as a resistance to the development of hypoxia -induced pulmonary hypertension. On the other hand, it is widely known that different models of hypertension can arise as a result of changes in endothelial function. The respect, one of the common causes of deregulation in endothelial vasodilator function have been associated with down-regulation of the NO synthesis and an increase in plasma levels of asymmetric dimethylarginine (ADMA) and homocysteine. Additionally, it is also known that NO production can be regulated by plasma levels of L-arginine as a result of the competition between nitric oxide synthase (NOS) and arginase. The objective of this study, was to determine the baseline concentrations of ADMA and homocysteine in llama, and to evaluate their effect on the arginase pathway and their involvement in the resistance to the development of altitude-induced pulmonary hypertension. METHOD: Lowland and highland newborn sheep and llama were investigated near sea level and at high altitude. Blood determinations of arterial blood gases, ADMA and homocysteíne are made and the effect of these on the arginase activity was evaluated. RESULTS: The basal concentrations of ADMA and homocysteine were determined in llama, and they were found to be significantly lower than those found in other species and in addition, the exposure to hypoxia is unable to increase its concentration. On the other hand, it was observed that the llama exhibited 10 times less arginase II activity as compared to sheep, and the expression was not induced by hypoxia. Finally, ADMA y Hcy, has no effect on the type II arginase pathway. CONCLUSION: Based on our results, we propose that low concentrations of ADMA and homocysteine found in llamas, the low expression of arginase type II, DDAH-2 and CBS, as well as its insensitivity to activation by homocysteine could constitute an adaptation mechanism of these animals to the hypoxia.

## Introduction

Hypoxic pulmonary vasoconstriction (HPV) in distal pulmonary arteries is an intrinsic vasoconstrictor response to low oxygen levels. Once the normoxic condition returns, the vasoconstrictor response returns to normal levels. If the hypoxic stimulus becomes chronic it leads to structural and functional maladaptation of the pulmonary arterial bed, characterized by pulmonary artery remodeling and vasoconstriction. This results in sustained pulmonary arterial hypertension (PAH) that is often accompanied by right ventricular hypertrophy, heart failure and eventually death (Giordano, 2005; Pe-aloza, 2012). Species that have adapted to life at high altitude and hypobaric hypoxia conditions in the Andean *altiplano* have existed for at least 10 million years. One such species is the llama (*Lama glama*), which developed efficient protective mechanisms to avoid maladaptation resulting from chronic hypoxia, such as a decreased hormonal vasoconstrictor response, an increased blood hemoglobin concentration, decreased cerebral O_2_ consumption, and increased Lactate Dehydrogenase activity (Llanos et al., 2003; Ebensperger et al., 2005). The llama possesses an attenuated cardiovascular response to hypoxia, which prevents the development of pulmonary hypertension and arteriolar muscle remodeling (Harris et al., 1982; Moraga et al., 2011). Contrary to llamas, newborn sheep gestated and born at high altitude have marked pulmonary hypertension compared to their counterparts (Llanos et al., 2011a).

Additionally, different models of hypertension can arise as a result of changes in endothelial function (Panza, 1997; Landmesser and Drexler, 2007). One of the most common causes of dysregulated endothelial vasodilator function is an alteration to the biochemical pathway that produces nitric oxide (NO) (Ignarro, 2002). Different models of hypertension are associated with down-regulation of the NO synthetic pathway and an increase in the plasma levels of asymmetric dimethylarginine (ADMA) and homocysteine. ADMA and homocysteine are inhibitors of nitric oxide synthase (NOS) which contributes to the pathogenesis of pulmonary hypertension (Arrigoni et al., 2003; Millatt et al., 2003; Wierzbicki, 2007; Lüneburg et al., 2016). Additionally, it has been reported that dimethyl-aminohydrolase (DDAH-2) (an enzyme that regulates the degradation of ADMA) and cystathionine β-synthase (CBS) (an enzyme that regulated the degradation of homocysteine) are target molecules due to the existence of polymorphisms in their genes that induce the dysregulation of their metabolism and, therefore, increase the risk of developing cardiovascular diseases (Zhang et al., 2014; Xuan et al., 2016).

NO production can be regulated by plasma levels of L-arginine as a result of the competition between NOS and arginase (Böger, 2004; Pernow and Jung, 2013). It has been suggested that arginase activity plays an important role in the pathogenesis of various pulmonary disorders (Maarsingh et al., 2008; Durante, 2013). For instance, an increase in arginase activity has been associated with several pulmonary and systemic hypertension models (Johnson et al., 2005; López et al., 2009; Chu et al., 2016). Nevertheless, there is no information available about the basal levels of ADMA and homocysteine in animals genetically adapted to life in high altitudes and, therefore, resistant to the development of pulmonary hypertension, such as the llama (*Lama glama*). Also, there is no information regarding the participation of the arginase signaling pathway and its regulation by ADMA and homocysteine in this model. Therefore, the aim of this study was to determine the baseline concentrations of ADMA and homocysteine in an animal model resistant to the development of pulmonary hypertension induced by hypoxia. We also evaluated the effect of ADMA and homocysteine on the arginase pathway and their involvement in the resistance to the development of altitude-induced pulmonary hypertension. We used sheep (Ovis aries) as a non-genetically adapted control group.

## Materials and methods

### Bioethics committee

All animal care, maintenance, procedures, and experimentation were performed in accordance with the United Kingdom's Animals (Scientific Procedures) Act 1986, and the American Physiological Society's Guiding Principles for Research Involving Animals and Human Being (American Physiologycal Society, 2002) and were reviewed and approved by the Faculty of Medicine Ethics Committee of the University of Chile.

### Animals

Lowland and highland newborn sheep (Ovis aries, *n* = 6) and llamas (*Lama glama, n* = 5) were studied near sea level (Santiago, 580 m, 710 mmHg barometric pressure. Lowland) and at high altitude (Putre, 3,600 m, 480 mmHg barometric pressure. Highland).

### Determination of arterial blood gases in lowland and highland newborn sheep and llamas

Newborns were submitted to a surgical procedure at 4–5 days old and studied at 7–10 days old. Animals were placed under general anesthesia with ketamine-diazepam association (10 mg kg^−1^ i.m. ketostop, Drag Pharma-Invectec, Santiago, Chile: 0.1–0.5 mg kg^−1^ i.m Diazepam, Laboratorio Biosano, Santiago, Chile) and additional local infiltration of 2% lidocaine (Dimecaíne, Laboratorio Beta, Santiago, Chile), polyvinyl catheters (1.2 mm i.d) were placed in the descending aorta and inferior vena cava via hindlimb artery and vein, exteriorized subcutaneously through the animal flank and kept in a pouch sewn onto the skin. Also, a Swan-Ganz catheter (Edwards Swan-Ganz 5 French, Baxter Healthcare Corporation, Irvine, CA, USA) was inserted into the pulmonary artery via an external jugular vein, exteriorized, and placed in a pouch around the neck of the animal. All vascular catheters were filled with a heparinized saline solution (500 IU heparin mL^−1^ in 0.9% NaCl) and plugged with a copper pin. Ampicillin 10 mg kg^−1^ i.v (Ampicilina, Laboratorio Best-Pharma, Santiago, Chile) was administered every 12 h while the animals were catheterized. The experiments commenced 3 days after surgery.

### Determination of arterio-pulmonary pressure

Pulmonary arterial pressure (PAP), systemic arterial pressure (SAP), and heart rate (HR) were recorded via a data acquisition system (PowerLab/8SP System and LabChart v7.0 Software; ADInstruments) connected to a computer. Cardiac output (CO) was determined with the thermodilution method by the injection of 3 ml of chilled (0°C) 0.9% NaCl into the pulmonary artery through the Swan-Ganz catheter connected to a CO computer (COM-2 model, Baxter). Mean PAP (mPAP), mean SAP (mSAP), and pulmonary (PVR) vascular resistances were calculated as described previously, Herrera et al. (2007).

### Determination of ADMA and homocysteine plasma concentrations

Plasma levels of ADMA (Enzo Life Sciences, Farmingdale, New York, USA) and homocysteine (Alpco Diagnostics, Salem, New Hampshire, USA) were measured by enzyme-linked immunosorbent assay (ELISA) according to the manufacturer's instructions.

### Enzyme preparations

The lungs were excised and frozen using liquid nitrogen and stored at −80°C until further use. The heart tissue was homogenized in a solution comprising of 10 mM Tris-HCl (pH 7.5), 0.1 mM PMSF, 1 mM DTT and 5 mM EDTA, and centrifuged at 5,000 g for 10 min at 4°C. The homogenates were centrifuged for 30 min at 5,000 g and 4°C and the supernatant was collected. Subsequently, the enzyme solution (supernatant) was separated by chromatography on a DEAE-cellulose column equilibrated with 5 mM Tris-HCl pH 7.5. The active fractions eluted with the washings of the column corresponded to arginase I, and the active fractions eluted at 0.10–0.15 M corresponded to arginase II (Venkatakrishnan et al., 2003).

The presence of arginase I and II, in the washing fraction and the eluted fraction, respectively, was confirmed by Western Blot. Finally, the active fractions were pooled and stored at −20°C.

### Extraction and quantification of DDAH-2 and arginase type II mRNA level by qRT- PCR

Total RNA was isolated using TRIZOL reagent (Invitrogen Corp., Carlsbad, CA, USA). RNA concentration and purity were assessed by Ultra Violet (UV) spectrophotometry (1.8 < A260/A280 < 2.0). RNA integrity was evaluated using electrophoresis. Reverse transcription reaction was carried out using 4 μg of total RNA and the First Strand complementary DNA (cDNA) Synthesis kit (Thermo Scientific, Boston, MA, USA).

To make standard curves, 1 μL of first-strand cDNA was amplified with AffinityScript cDNA Synthesis Kit and quantification of Polymerase Chain Reaction (PCR) products were used for plotting standard curves.

Quantitative real-time PCR was carried out using a HT-7500 thermo- cycler (Applied Biosystems) and using Brilliant III Ultra-Fast SYBR® Green QPCR Master Mix. program PCR was of 95°C for 3 min, followed by 40 cycles of 95°C for 30 s and 60°C for 30 s. GAPDH was used as an internal control.

DDAH-2, CBS and eNOS mRNA level was normalized with GAPDH (Gliceraldehído-3-fosfato deshidrogenasa) mRNA to compensate for variations in initial RNA amounts. Normalization was carried out by dividing the logarithmic value of DDAH- 2, CBS and eNOS by the logarithmic value of GAPDH.

### Arginase activity measurements

Specific arginase activity was determined in lung tissues based on urea production from L-arginine using a colorimetric method with α-isonitrosopropiopheonone (Archibald, 1957). Briefly, 50 μl of the fractions obtained from DEAE-cellulose were incubated in 30 mM Tris-HCl (pH 7.5) containing 100 mM L-arginine at 37°C for 10 min. The reaction was stopped by the addition of 1 ml of an acid mixture containing 9% (v/v) of H_3_PO_4_ and 23% (v/v) of H_2_PO_4_. To evaluate the effect of homocysteine and ADMA on arginase activity, a specific arginase activity assay was performed in the presence of physiological concentrations of ADMA and homocysteine (0.5, 1.0, 3.0, 6.0, and 10.0 μM) in a 50 mM Tris-HCl solution (pH 7.5).

### Data analysis and statistics

All statistical analyses were performed using GraphPad Prism 5.0 software. The mean values and standard error (SE) were calculated for each parameter. The normality of quantitative variables was established using the Kolmogorov–Smirnov test. The statistical validity of the difference across all testing conditions was established using analysis of variance (ANOVA) of one factor, and ANOVA for repeated measures was performed for differences within the group. Pearson's correlation was also performed. Linear regression analysis was carried out to establish the association between arginase activity (dependent variable) and the other variables. The significance level was established at *p* < 0.05.

## Results

A decrease in PaCO_2_, PaO_2_, and SaO_2_ values was observed at high altitude conditions in both sheep and llamas. However, newborn llama SaO_2_ values were higher at high altitude (Table 1). The biggest difference observed between the two species was the higher hemoglobin oxygen saturation in newborn llama compared to newborn sheep at high altitude. This difference is explained by the higher hemoglobin oxygen affinity of llamas compared to sheep (Moraga et al., 1996).

**Table 1:** Arterial blood gases in lowland and highland newborn sheep and llamas (*n* = 5).

**Sheep**		**Llamas**	
**Lowland**	**Highland**	**Lowland**	**Highland**
**PaCO**_2_ **(mmHg)**
37 ± 1	^*^32 ± 2	37 ± 1	^*^32 ± 2
**PaO**_2_ **(mmHg)**
78 ± 3	^*^41 ± 4	94 ± 3	52 ± 4
**SaO**_2_ **(%)**
95 ± 1	^*^66 ± 4	97 ± 2	92 ± 2

* *Values represent the mean ± SE (n = 5 per group). p < 0.01*.

### Newborn llamas are resistant to the development of hypoxia-induced pulmonary hypertension

**Figure 1:**
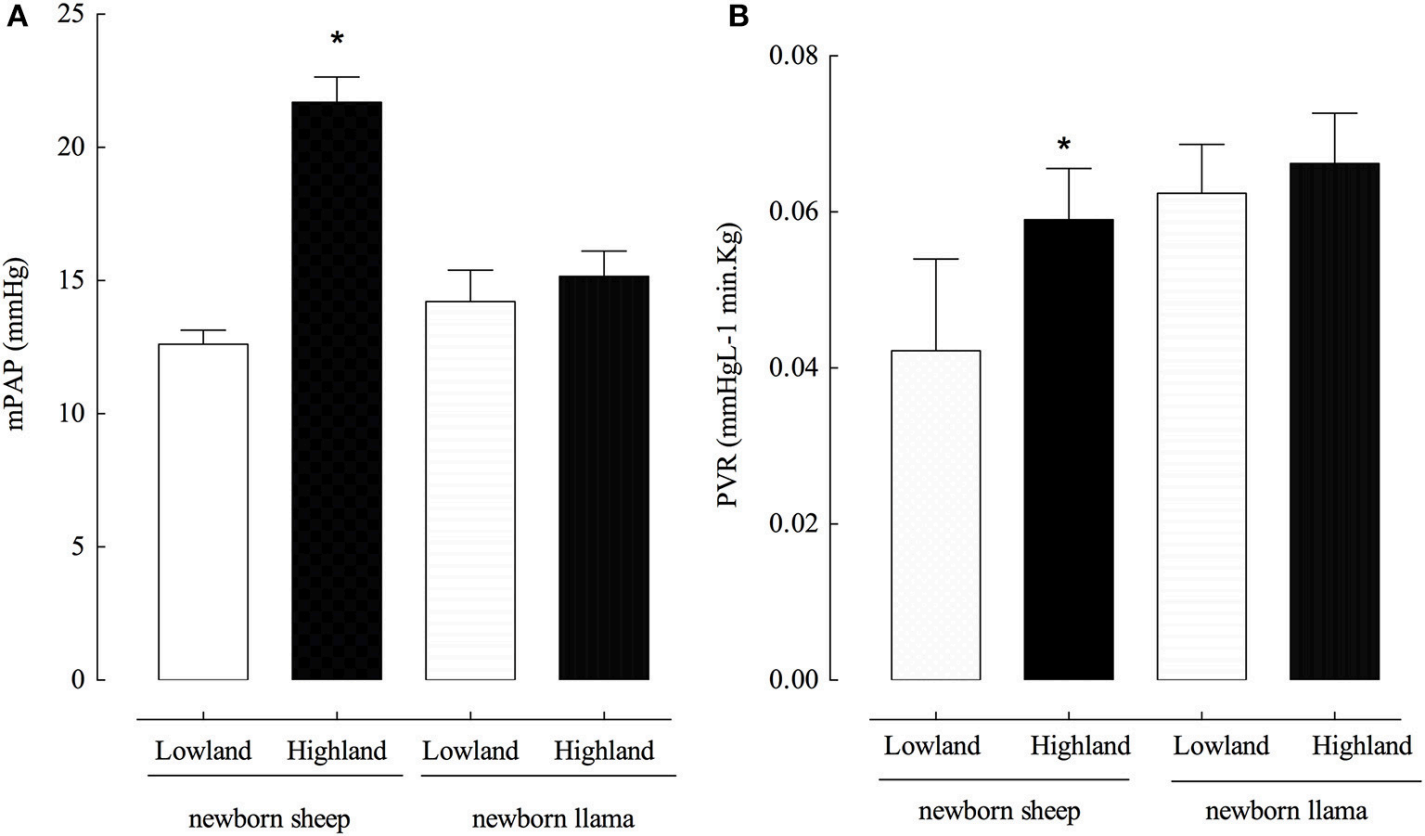
Pulmonary arterial pressure (PAP) and pulmonary vascular resistance (PVR) in newborn sheep and llamas. **(A)** A significant difference was found between the PAP of lowland newborn sheep vs. highland newborn sheep (^*^p < 0.01). No significant differences were observed in the PAP of llamas between these two conditions (n.s). **(B)** A significant difference was found between the PVR of lowland newborn sheep vs. highland newborn sheep (^*^p < 0.01). No significant differences were observed in the PVR between newborn llamas in these two conditions (n.s). ^*^(Llama *n* = 5; sheep *n* = 6).

Newborn llamas do not present significant differences in mPAP (14 ± 1 and 15 ± 0.5 mmHg) between lowland and highland conditions (Figure 1A). No modifications were observed in PVR in newborn llamas at lowland vs. highland (Figure 1B). On the contrary, newborn sheep had an increased mPAP (12 ± 0.6 mmHg) at lowland compared to highland levels (21 ± 2.0 mmHg) (Figure 1A). Additionally, highland newborn sheep exhibited increased PVR values compared to lowland newborn sheep (Figure 1B). No significant difference were observed in PVR between both species at lowland and highland (*p* > 0.05).

### Llamas have low ADMA and homocysteine concentrations that do not increase when exposed to hypoxia

**Figure 2:**
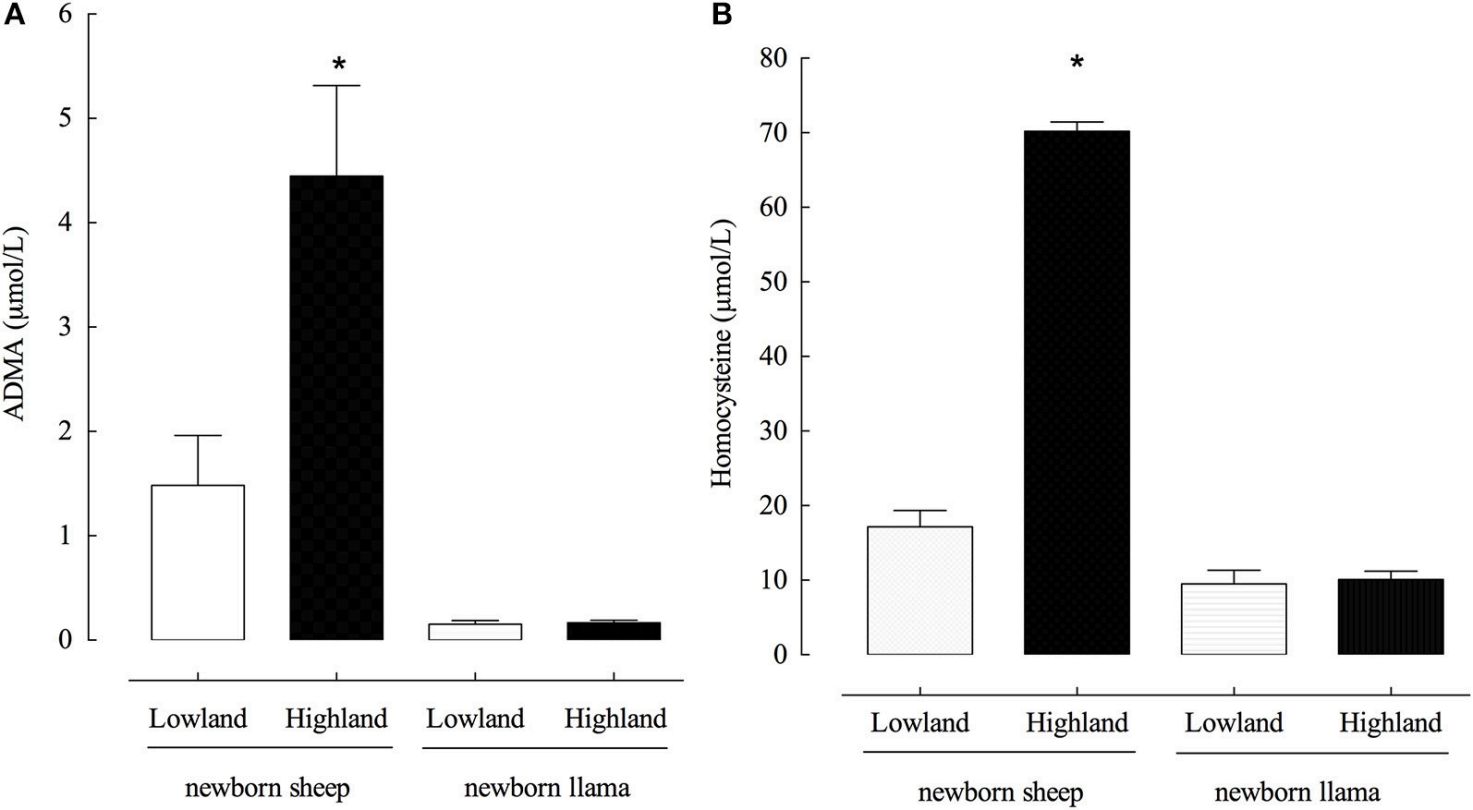
Plasma concentrations of ADMA and homocysteine in newborn llamas and sheep. **(A)** Plasma concentration of ADMA. A significant difference was found between lowland and highland newborn sheep (^*^p < 0.01). **(B)** Plasma concentration of Homocysteine. A significant difference was found between lowland and highland newborn sheep (^*^p < 0.01). No significant differences were observed between ADMA and homocisteine concentrations was found between lowland and highland newborn llama (n.s).

To evaluate the role of ADMA and homocysteine in the development of pulmonary hypertension, we measured the basal concentrations of ADMA and homocysteine in the plasma of lowland and highland newborn sheep and llamas. Llamas had significantly lower plasmatic ADMA concentrations compared to sheep, both in lowland and highland newborn animals. In fact, ADMA concentrations in lowland conditions were 1.48 μM in sheep and 0.153 μM in llamas, approximately 10 times lower (Figure 2A).

On the contrary to the expected results, exposure to hypoxia did not increase ADMA concentrations in llamas, whereas hypoxic conditions induced a significant increase in the ADMA concentration in sheep, from 1.4 to 4.45 μM (approximately a 4x increase) (Figure 2A).

The same phenomenon was observed with the concentration of homocysteine which was significantly lower in llamas compared to sheep, and hypoxia was incapable of producing an increase (Figure 2B). In fact, sheep had higher concentrations of homocysteine and hypoxia significantly increased the concentration from 17.32 to 70.4 μM (approximately a 4x increase).

### Llamas have low DDAH-2 and CBS expression levels and which are unaffected by hypoxia

**Figure 3:**
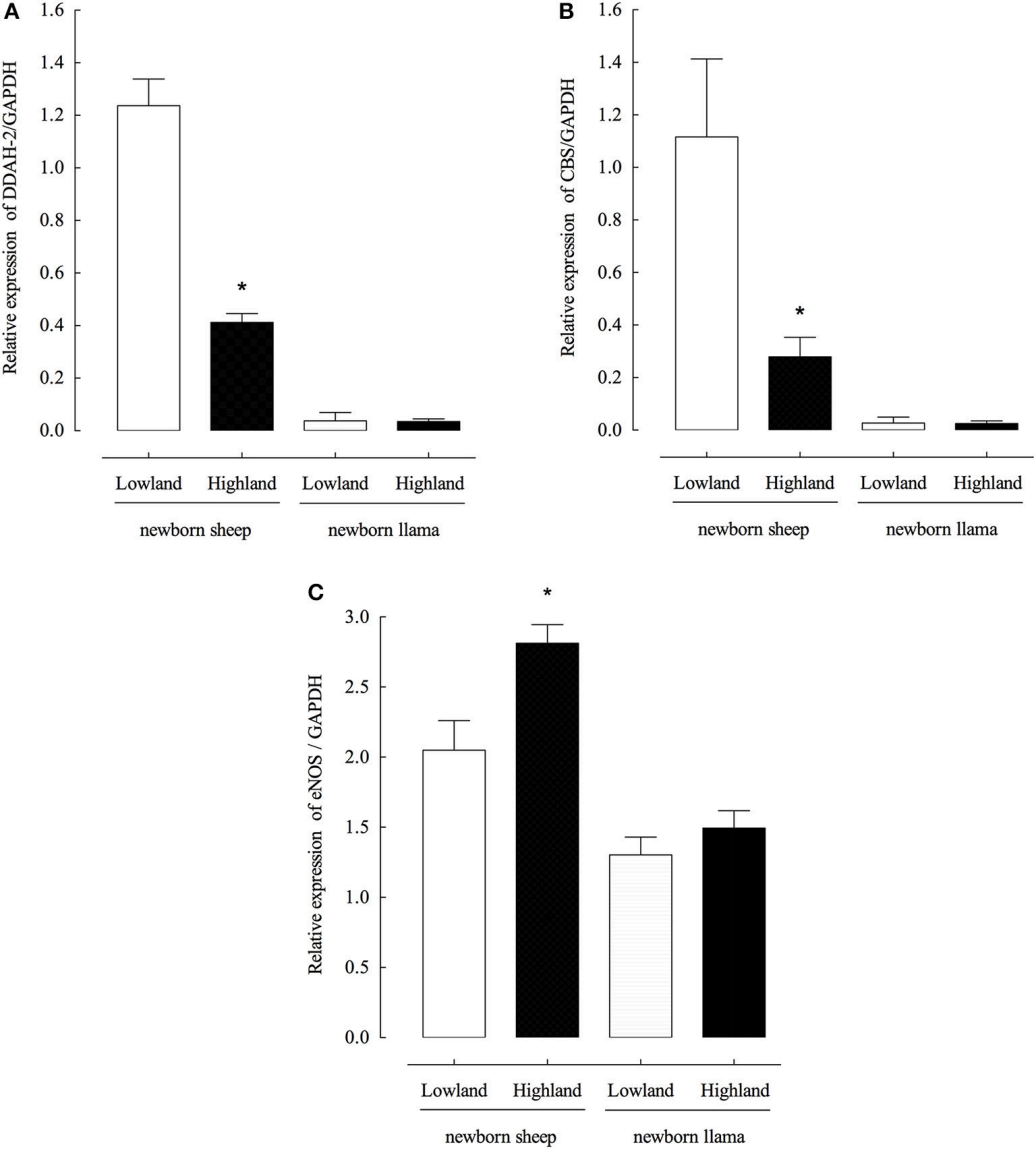
Expression of DDAH-2, CBS and eNOS genes from the lung parenchyma of newborn llamas and sheep. **(A)** Quantitative PCR of DDAH-2 **(B)** Quantitative PCR of CBS **(C)** Quantitative PCR of eNOS. Gene expression was measured by real-time PCR; data were normalized to GAPDH levels. (*n* = 6 per group, ^*^p < 0.001 group).

To evaluate the relationship between the observed concentrations of ADMA and homocysteine and the expression levels DDAH-2 (dimethylarginione dimethyaminohydrolase-1) and CBS (cystathionine-β-synthase), enzymes responsible for the regulation of the endogenous concentrations of ADMA and homocysteine, respectively, the expression levels of DDAH-2 and CBS mRNAs from the lung parenchyma were measured. Low concentrations of DDAH-2 and CBS mRNA from llama lung parenchyma were detected under conditions of normoxia and hypoxia (Figures 3A,B), unlike sheep, a significant decrease in expression levels of both genes was observed during hypoxia.

The expression levels of endothelial NOS were also evaluated and it was determined that in the llama there is not significant increase in eNOS expression levels during hypoxia conditions (Figure 3C).

### Llamas have low levels of type II arginase activity which is not up-regulated during hypoxia

**Figure 4:**
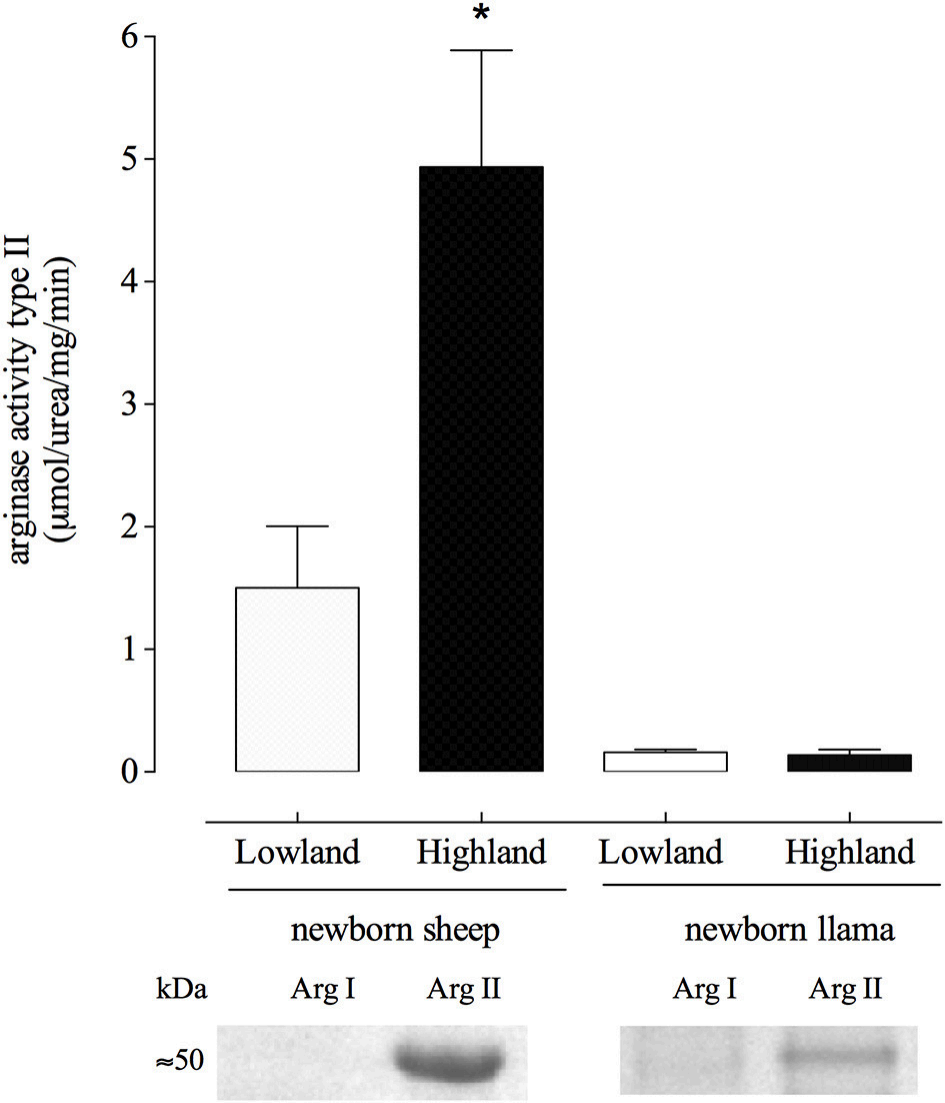
Arginase type II activity from lung parenchyma of newborn llamas and sheep. A significant difference was found between newborn sheep in highland vs. lowland conditions. (^*^p < 0.01). No significant differences were observed in arginase activity between these two conditions in llamas (n.s). (The western blots correspond to the eluted fraction of DEAE-cellulose chromatography).

An increase in the expression and activity of arginase has been related to the development of hypertension in different models. Therefore, we measured arginase type II activity in homogenized lungs from newborn sheep and llamas at lowland and highland (Figure 4).

Llamas exhibited 10 times less arginase II activity compared to sheep which was not induced by hypoxia (Figure 4). Furthermore, arginase II activity remained at similar levels in llamas at lowland and highland.

Additionally, hypoxia induced a significant increase of arginase type II activity by approximately 5 times in newborn sheep.

### Arginase type II in llamas is insensitive to activation by homocysteine

**Figure 5:**
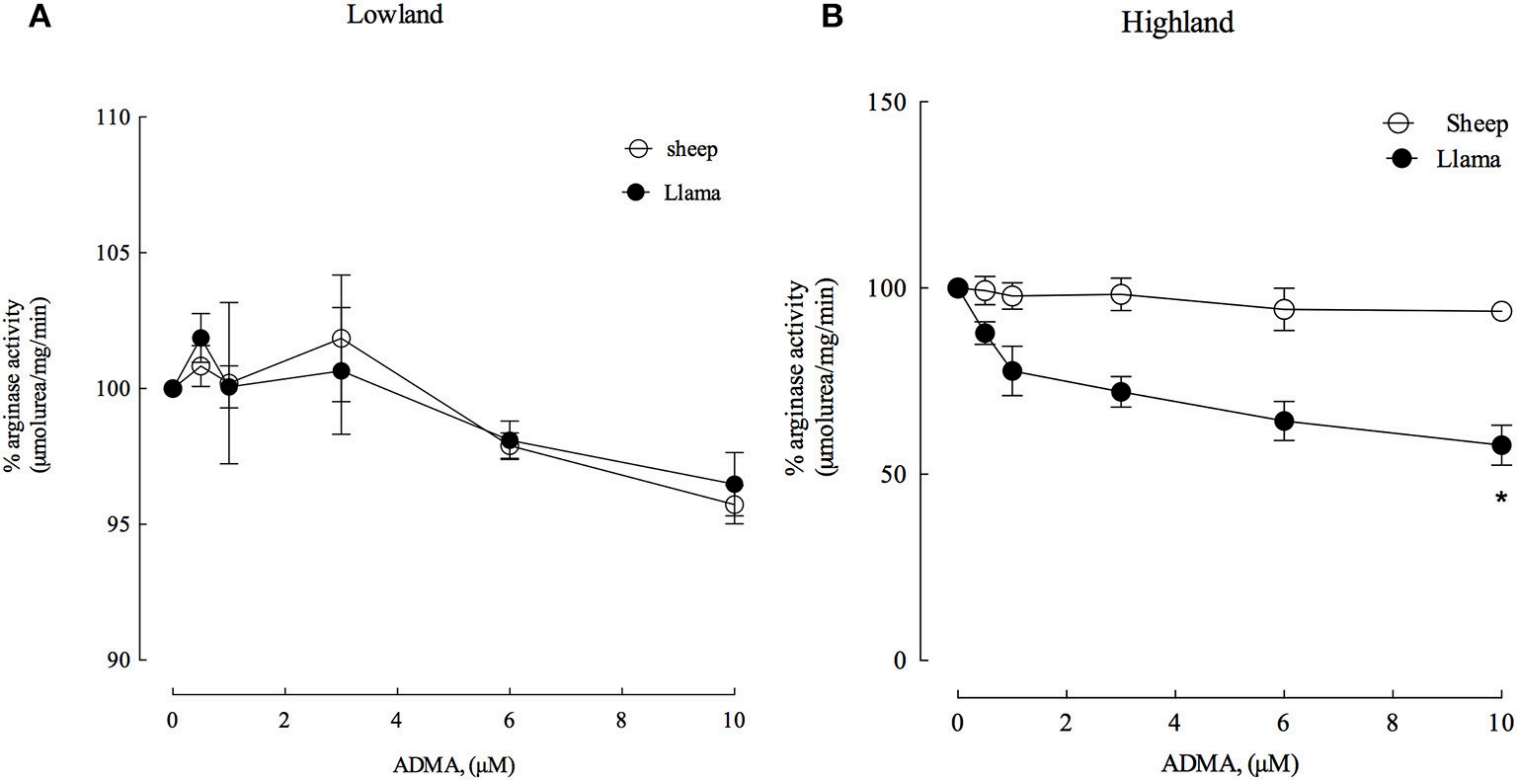
Effect of increasing ADMA concentrations on arginase type II activity from lung parenchyma in newborn llamas and sheep. **(A)** The effect of ADMA on arginase type II activity in lowland conditions. No significant differences were observed in arginase activity between sheep and llamas. **(B)** The effect of ADMA on arginase type II activity in Highland conditions. A significant difference was found between newborn sheep and llamas. ^*^Values represent the mean ±SE (*n* = 5 per group). ^*^p < 0.01, Llamas vs. sheep.

**Figure 6:**
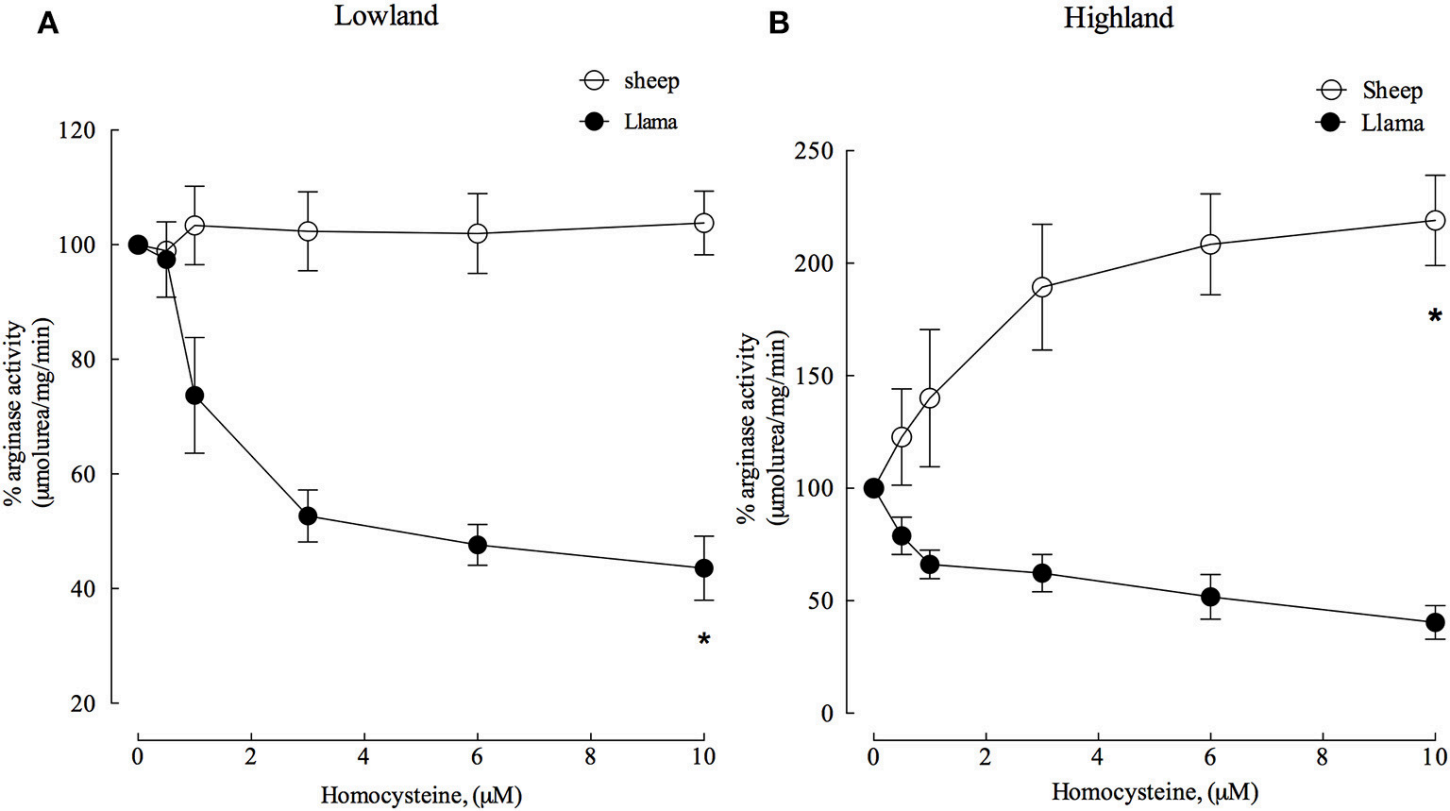
Effect of increasing homocysteine concentrations on arginase type II activity from lung parenchyma in newborn llamas and sheep. **(A)** The effect of homocysteine on arginase type II activity in lowland conditions. A significant difference was observed in arginase activity between sheep and llamas. **(B)** The effect of homocysteine on arginase type II activity in Highland conditions. A significant difference was found in between newborn sheep and llamas. ^*^Values represent the mean ±SE (*n* = 5 per group). ^*^p < 0.01, Llamas vs. sheep.

ADMA and homocysteine have been described as markers for cardiovascular risk and previous results have shown that ADMA and homocysteine have an activation effect on arginase type II from hypoxia-induced hypertensive rats (López et al., unpublished data). Therefore, we evaluated the effect of these metabolites on arginase type II activity from the lung parenchyma of newborn llamas and sheep (Figures 5, 6). ADMA was a poor inhibitor of arginase type II activity in newborn llamas and sheep at lowland (Figure 5A) and highland (Figure 5B). However, increased inhibition of arginase type II activity by ADMA was observed in hypoxic conditions in llamas, demonstrating an inhibition of approximately 25% at physiological concentrations of ADMA (1 μM) and reaching a maximum inhibition level of 40% at higher concentrations (10 μM). Additionally, arginase type II activity of newborn llamas in normoxic conditions was not affected by ADMA (Figures 5A,B).

Experiments with homocysteine demonstrated that arginase type II activity in sheep was not affected at lowland. However, homocysteine (4 μM) was an important inhibitor and reduced enzyme activity by approximately 50% (Figure 6A) in llamas.

In highland conditions, homocysteine was an important activator of arginase type II in sheep, increasing its activity with increasing concentrations. On the contrary, arginase type II was inhibited in llamas by approximately 50% with 6 μM homocysteine (Figure 6B).

## Discussion

Llamas are genetically adapted to live in hypobaric hypoxia conditions at high altitudes. Among the physiological adaptations of the adult llama that allow it to live under conditions of oxygen limitation at altitude are: an increased blood hemoglobin concentration, low P_50_, high muscle myoglobin concentration, a more efficient O_2_ extraction at tissue levels, and high lactic dehydrogenase activity. Furthermore, the adult llama avoids pulmonary arterial hypertension and cardiac remodeling, among other adverse effects, by having less muscularized pulmonary arterioles than the adult sheep (Llanos et al., 2011b). Together, these adaptations allow the llama to adapt to life at high altitudes. In agreement with the adaptations of the pulmonary circulatory system previously described, ours results showed that newborn llama at lowland or highland do not show hypertension in pulmonary arteries when calculating the PVR using Ohm's law (Herrera et al., 2007). We observed that the lack of PVR modifications in newborn llama is explained by the maintenance in the cardiac output, similar to that described by Herrera et al. (2008). In contrast, in newborn sheep the higher mPAP and PVR observed at high altitude is explained by an increased cardiac output in newborn sheep. The unaltered PVR in newborn llamas can be explained by their lack of hypertrophic pulmonary vascular remodeling and pulmonary hypertension (Llanos et al., 2011b) by a proposed blunting in this response.

Additionally, studies performed by Herrera et al. (2008) showed that there is an increased pulmonary CO production compared to NO production in newborn llamas. CO is an alternative vasodilator synthesized by the HO- carbon monoxide system that is activated during chronic hypoxia because the relative increase in NO production is not sufficient to counteract the increase in PAP at high altitude. Thus, these authors proposed that activation of the HO-carbon monoxide system induces an adaptation mechanism in these animals during birth that would protect their vascular musculature against the effects of chronic hypoxia and would explain their resistance to the development of pulmonary hypertension. In contrast, the newborn sheep present pulmonary vascular remodeling (Llanos et al., 2011b) and produce hypoxic pulmonary constrictor responses. These observations support our results, since we did not observe a significant increase in eNOS levels in llamas (Figure 3C), which would indicate an insufficient amount of NO concentrations and, therefore, reinforce the importance of the activation of the HO-carbon monoxide system in these animals.

In this study, we determined the concentrations of ADMA and homocysteine for the first time in an animal genetically adapted to live at high altitudes. Data showed that llamas had a lower ADMA and homocysteine concentration compared to sheep and other described species (Böger et al., 2000; Lüneburg et al., 2016; Figure 2A). It is widely known that ADMA and homocysteine are risk factors for cardiovascular diseases (Wierzbicki, 2007; Böger et al., 2009). We have recently described that hypoxia induces an increase in the concentration of homocysteine and ADMA in hypoxia-induced hypertensive rats (López et al., unpublished). Therefore, based on our study, we hypothesize that low concentrations of ADMA and homocysteine ensure a baseline level of NO synthesis, which together with activation of CO production, results in an adaptation system that allows llamas to live in hypobaric hypoxia conditions without developing pulmonary hypertension.

One explanation for the low levels of ADMA and homocysteine found in llamas could be due to the low expression of key enzymes for the regulation of their synthesis. According to currently available literature, key enzymes in the metabolism of methionine, and therefore in the production of ADMA and homocysteine, have been described as “target molecules” due to the presence of polymorphisms that lead to a deregulation in their metabolism and to the development of cardiovascular diseases. Among these enzymes are cystathionine β-synthase (CBS) (Zhang et al., 2014), methylenetetrahydrofolate reductase (MTHFR) (Yang et al., 2014; Heifetz and Birk, 2015) and dimethyl-aminohydrolase (DDAH-2) (Xuan et al., 2016). The expression and activity levels of these enzymes in llamas and whether they play an important role as therapeutic targets in the hypertensive processes induced by hypoxia remains unknown. Our results show that low expression levels of arginase type II mRNA, associated to the low concentrations of ADMA and Hcy found in llamas, suggest that the NO- arginase pathway is not involved in the resistance to the development of altitude- induced pulmonary hypertension (Figures 2, 3).

Additionally, hypoxia did not increase ADMA concentrations which could suggest that, unlike other species, the gene for dimethyl-aminohydrolase-2 (DDAH-2) in llamas does not contain hypoxia response elements such as the hypoxia-inducible factor (HIF-1α) (Pekarova et al., 2015). Contrary to what could be expected in llamas, we found a significant reduction in DDAH-2 expression in rats intolerant to the development of hypoxia-induced hypertension, which could explain the observed increase in ADMA.

Newborn llamas express at least 10 times less arginase type II which is not activated by hypoxia (Figure 3). It is widely known that one of the causes of endothelial dysfunction is decreased NO synthesis, which could be explained by a reduction in the concentration of L-arginine required for its synthesis. For this reason, it was shown that an increase in the expression of arginase would be involved in the development of hypertension, since it would compete with eNOS for the use of L- arginine (Durante et al., 2007; Pernow and Jung, 2013). Based on our results, we propose that the lower arginase type II levels leads to greater bioavailability of L-arginine for the synthesis of NO (Figure 4) which would explain the resistance to the development of hypertension induced by hypoxia in newborn llamas. In other words, the lower expression of arginase type II in these animals may constitute a mechanism for adaptation at geographical altitudes.

Finally, both ADMA and homocysteine are inhibitors of arginase type II in llamas in both lowland and highland conditions (Figures 5, 6). On the contrary, homocysteine from sheep is an activator of arginase (Figure 6B). In agreement with previous studies, we found that homocysteine has the same activating effect on arginase II from hypoxia-induced hypertensive rats (López et al., unpublished data). Therefore, the inability of homocysteine and ADMA to activate arginase type II in llamas could represent a protection mechanism in llamas, which would prevent the increased expression of arginase type II in hypoxic conditions in these animals (Figure 6B).

**Figure 7:**
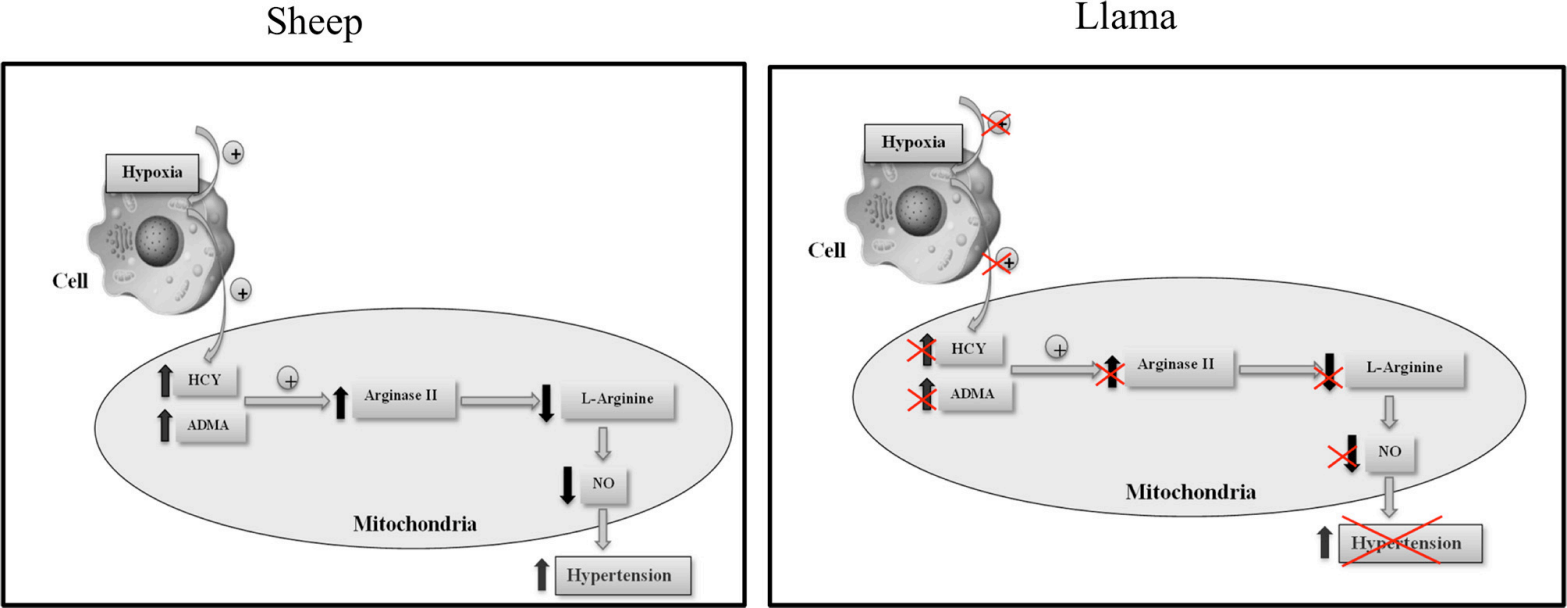
Proposed mechanism for the participation of ADMA, homocysteine and arginase type II in regulating the development of hypoxia-induced hypertension in llamas.

Based on our results, we propose that low concentrations of ADMA and homocysteine found in llamas, and also the low arginase type II levels, could constitute an adaptation mechanism that these animals present when faced with hypoxic conditions by impeding the reduction in L-arginine, and thus ensuring baseline synthesis of NO during hypoxia (Figure 7).

## Author contributions

EU supervises the overall the study. Contributed to sample and data collections. The authors drafted the report. All authors contributed to the interpretation of the results, critical revision of the manuscript and approved the final manuscript.

### Conflict of interest statement

The authors declare that the research was conducted in the absence of any commercial or financial relationships that could be construed as a potential conflict of interest.
